# Ergogenic Benefits of β‐Hydroxy‐β‐Methyl Butyrate (HMB) Supplementation on Body Composition and Muscle Strength: An Umbrella Review of Meta‐Analyses

**DOI:** 10.1002/jcsm.13671

**Published:** 2025-01-10

**Authors:** Mohammad Vesal Bideshki, Mehrdad Behzadi, Mehrdad Jamali, Parsa Jamilian, Meysam Zarezadeh, Bahram Pourghassem Gargari

**Affiliations:** ^1^ Student Research Committee Tabriz University of Medical Sciences Tabriz Iran; ^2^ Department of Biochemistry and Diet Therapy, School of Nutrition and Food Science Tabriz University of Medical Sciences Tabriz Iran; ^3^ Student Research Committee, School of Nutrition and Food Sciences Shiraz University of Medical Sciences Shiraz Iran; ^4^ School of Medicine Keele University Stafforshire UK; ^5^ Faculty of Nutrition and Food Science Tabriz University of Medical Sciences Tabriz Iran; ^6^ Nutrition Research Center, Department of Biochemistry and Diet Therapy, Faculty of Nutrition and Food Sciences Tabriz University of Medical Sciences Tabriz Iran

**Keywords:** body composition, fat‐free mass, HMB, muscle mass, umbrella meta‐analysis, β‐hydroxy‐β‐methyl butyrate

## Abstract

**Background:**

β‐Hydroxy‐β‐methyl butyrate (HMB) is a metabolite of the amino acid leucine, known for its ergogenic effects on body composition and strength. Despite these benefits, the magnitude of these effects remains unclear due to variability among studies. This umbrella review aims to synthesize meta‐analyses investigating the effects of HMB on body composition and muscle strength in adults.

**Methods:**

A comprehensive literature search was conducted in Scopus, PubMed and Web of Science without date or language restrictions until August 2024. The study protocol was registered at Prospero (No. CRD42023402740). Included studies evaluated the effects of HMB supplementation on body mass, fat mass (FM), fat‐free mass (FFM), muscle mass and performance outcomes. Effect sizes (ESs) and 95% confidence intervals (CIs) were calculated, and a random‐effects model was used for meta‐analysis. Standard methods assessed heterogeneity, sensitivity and publication bias. The methodological quality of included studies was assessed using the AMSTAR2 tool.

**Results:**

Eleven studies comprising 41 data sets were included, with participants aged 23–79 years. HMB supplementation significantly increased muscle mass (ES: 0.21; 95% CI: 0.06–0.35; *p* = 0.004), muscle strength index (ES: 0.27; 95% CI: 0.19–0.35; *p* < 0.001) and FFM (ES: 0.22; 95% CI: 0.11–0.34; *p* < 0.001). No significant changes were observed in FM (ES: 0.03; 95% CI: −0.04 to 0.35; *p* = 0.09) or body mass (ES: 0.09; 95% CI: −0.06 to 0.24; *p* = 0.22). The quality assessment revealed that five studies were of high quality, three were of low quality and three were of critically low quality.

**Conclusions:**

HMB supplementation may benefit individuals experiencing muscular atrophy due to physiological conditions, particularly enhancing muscle mass and strength without significant changes in fat mass or body weight.

## 1 Introduction

β‐Hydroxy‐β‐methyl butyrate (HMB) is a natural compound that is produced in the body during the metabolism of the branched‐chain amino acid leucine. A number of potential health benefits, especially on body composition, have made it popular in the fitness and sports nutrition industry [1]. Currently, leucine and its metabolites have received a great deal of attention due to their anabolic properties [2]. Among these metabolites, HMB is likely to have the greatest anabolic and anticatabolic effects [3]. The findings of research have demonstrated that HMB enhances the synthesis of muscle proteins (up to 70%), reduces the breakdown of muscle proteins (up to 57%) and improves the stability of muscle membranes, resulting in improvement of muscle quality [4]. In addition to augmenting muscle glycogen storage, HMB increases mitochondrial biogenesis, which, in turn, boosts oxidative function, increasing aerobic capacity [5, 6]. Additionally, these benefits could be attributable to an increase in lean body mass (LBM) and/or fat‐free mass (FFM) [7] and a reduction in fat mass (FM) [8]. Beyond its role in improving body composition and muscle strength, muscle mass plays a critical role in cardiometabolic disease prevention. Recent research has demonstrated that higher levels of muscle mass are associated with improved metabolic profiles, including enhanced insulin sensitivity and lipid metabolism, which are key factors in reducing the risk of conditions such as Type 2 diabetes and cardiovascular disease [9]. Given the increasing prevalence of these diseases, interventions that promote muscle mass, such as HMB supplementation, may be particularly beneficial in mitigating these risks, especially in populations prone to muscle atrophy [10].

However, a number of studies have investigated the effects of HMB supplementation on muscle strength in various populations, such as athletes, older adults and individuals participating in resistance training [11, 12]. By enhancing the rate of muscle repair and reducing muscle damage, this supplementation can contribute to greater gains in strength over time [13]. To achieve this effect, several mechanisms have been proposed, including the use of increasing the efficiency of oxidative metabolism, a process associated with the generation of increased muscle strength [10, 14]. Although many scientific studies have been conducted to support the ergogenic effects of HMB on strength and body composition in humans, the results show both improvements and no change in strength and body composition with HMB supplementation [8, 15, 16, 17, 18].

Overall, various systematic reviews and meta‐analyses provide contradictory conclusions regarding HMB's effectiveness in increasing body composition and strength, considering the differences in the studied populations from athletes to elderly individuals. Hence, we intend to conduct an umbrella meta‐analysis to clarify the health benefits of HMB supplementation on body composition and muscle strength in a variety of age groups and health conditions.

## 2 Methods

We conducted this umbrella meta‐analysis in accordance with the Preferred Reporting Items for Systematic Reviews and Meta‐Analyses (PRISMA) recommendations [19]. The protocol of the study was registered in the PROSPERO database (registration code: CRD42023461929).

### 2.1 Search Strategy and Study Selection

For relevant literature published up to August 2024, we searched electronic databases including Scopus, EMBASE, Web of Science and PubMed. Using Medical Subject Headings (MeSH) as search terms and keywords, the following search strategy was designed: (‘β‐hydroxy β‐methylbutyrate’ OR ‘beta‐Hydroxy beta‐methylbutyric acid’ OR ‘beta Hydroxy beta methylbutyric acid’ OR ‘3‐hydroxyisovaleric acid’ OR HMB OR ‘beta methylbutyrate’ OR ‘b‐hydroxyb‐methylbutyrate’ OR ‘hydroxy methylbutyrate’) AND (‘meta‐analysis’ OR ‘meta analysis’ OR ‘meta review’ OR ‘meta’). Only studies in English were considered eligible.

### 2.2 Inclusion and Exclusion Criteria

According to the population/intervention/comparison/outcomes (PICOS), the following relevant studies were selected: P (adults older than 18 years old), I (HMB supplementation), C (placebo or control group), O (body composition and muscle strength) and study design (meta‐analyses of randomized clinical trials [RCT]) (Table 1). We excluded studies with the following criteria: (1) controlled clinical trials, (2) quasiexperimental studies, (3) observational studies, (4) in vivo studies, (5) ex vivo studies, (6) in vitro studies and (7) systematic reviews.

**TABLE 1:** PICOS criteria for inclusion and exclusion of studies.

Parameter	Criteria
Participant	Adults
Intervention	HMB supplementation
Comparator	Placebo or control group
Outcomes	Body composition and muscle strength
Study design	Meta‐analyses of randomized clinical trials

Abbreviation: HMB: β‐hydroxy‐β‐methyl butyrate.

### 2.3 Research Objectives

The study's objectives were to determine the impact of HMB supplementation on body composition, including body mass, FM, FFM, muscle mass and performance outcomes for muscle strength, such as handgrip strength, leg strength and bench press.

### 2.4 Methodological Quality Assessment

To assess the methodological quality of the included articles, two reviewers (M.V.B. and M.B.) independently completed the Assessment of Multiple Systematic Reviews 2 (AMSTAR2) questionnaires [20]. The questionnaire includes 16 items in total to answer ‘Yes’ or ‘Partial Yes’ or ‘No’ or ‘Not a Meta‐Analysis’. The AMSTAR2 checklist was classified into ‘critically low quality’, ‘low quality’, ‘moderate quality’ and ‘high quality’. The online version of this questionnaire (https://amstar.ca/Amstar_Checklist.php) was used to assess this evaluation more accurately and reliably. Discrepancies were resolved following a discussion with a third reviewer (M.Z.).

### 2.5 Study Selection and Data Extraction

Following the removal of duplicate publications, the titles and abstracts of the publications were screened by two researchers (M.V.B. and M.B.) independently in accordance with the eligibility criteria. Interrater agreement was measured using Cohen's kappa coefficient, which yielded a value of 0.82 (*p* value < 0.01), indicating almost perfect agreement between the reviewers. This approach ensured a consistent and objective assessment of the included studies [21]. As a second screening step, the full texts were reviewed for the remaining articles. An Excel spreadsheet was then created with the information extracted from the included studies. The following data were extracted: study location, year of publication, first author's name, sample size, gender, participants' age, type of HMB, supplementation duration, HMB dosage, health condition, effect sizes (ESs) and 95% confidence intervals (CIs) for body mass, FM, FFM, muscle mass and performance outcomes for muscle strength, such as handgrip strength, leg strength and bench press.

### 2.6 Data Synthesis and Statistical Analysis

Based on the ESs and 95% CIs of each included study, we calculated the overall ES. To obtain the pooled results, a random‐effects model with the restricted maximum likelihood (REML) method was used. Cochrane's *Q* test and *I* ^2^ statistics were used to determine the between‐study heterogeneity. If the *I* ^2^ value was > 50% or the *p* value was < 0.1, the data were considered heterogeneous. Considering the differences between standard and weighted mean differences (SMD and WMD), the data for each outcome were analysed separately. Furthermore, subgroup analyses were performed based on the number of included studies, dosage, duration, age, control type, quality of the studies and type of ES (WMD/SMD) to find potential sources of heterogeneity and to present ESs across various subgroups. Using a sensitivity analysis, we excluded studies one by one from the analysis and assessed how their exclusion affected the pooled estimate. Formal Egger's [22] and Begg's [23] tests and visual inspection of the funnel plots [24] were conducted to assess the small‐study effect. Once publication bias was detected, the trim‐and‐fill method was used to simulate a model considering publication bias. All statistical analyses were conducted using STATA for Windows Version 16.0 (Stata Corporation, College Station, TX, United States). The level of significance was determined as *p* < 0.05.

## 3 Results

### 3.1 Literature Review

**FIGURE 1:**
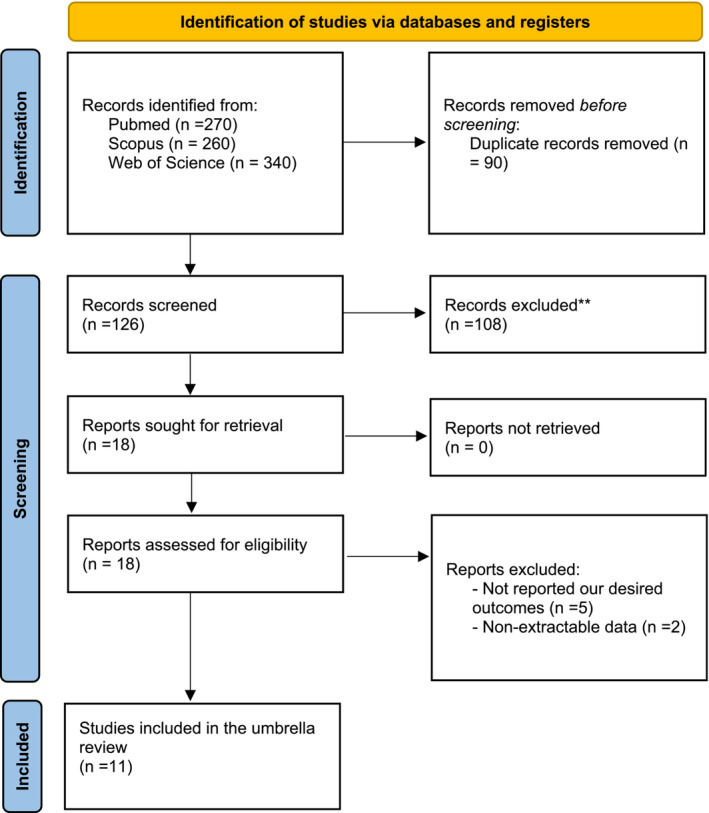
PRISMA flow chart of the study illustrating the study selection process.

First, we retrieved articles by searching databases. Second, 126 studies relevant to the effect of HMB on body composition and muscle strength outcome remained after deduplication. Then, after evaluating the titles and abstracts, 108 articles were excluded. Next, 18 full‐text articles were selected for further review and evaluation. Upon analysis of the full texts of these 18 studies, seven articles were excluded for one or more of the following reasons: not reporting our desired outcomes (*n* = 5) [25, 26, 27, 28, 29] and nonextractable data (*n* = 2) [30, 31] (Figure 1). Finally, a total of 11 studies with 41 data sets were regarded as eligible for the umbrella review.

### 3.2 Methodological Quality Assessment

Table S1 presents the findings of the quality assessment of meta‐analyses according to the AMSTAR2 questionnaire. Five studies obtained a high ranking in terms of quality [13, 17, 32, 33, 34]. Three studies were of low quality [35, 36, 37]. Three studies were of critically low quality [18, 38, 39].

### 3.3 Characteristics of the Included Meta‐Analyses

The characteristics of 11 meta‐analyses with 41 data sets are presented in Table 2. Among them, three articles were conducted in China [13, 34, 37], two in the United States [38, 39], one in Canada [32], one in Spain [36], one in Chile [17], one in New Zealand [18], one in Australia [35] and one in the United Kingdom [33]. All included studies were meta‐analyses of RCTs published from 2003 to 2022. Eight studies, including 11 data sets, were conducted to assess the effects of HMB on FM. Four studies including five data sets investigated the impact of HMB supplements on body mass. A total of five studies including five data sets examined changes in muscle mass as a dependent variable in response to the administration of HMB. Five studies, including eight data sets, have been conducted to assess the effects of HMB on FFM, and a total of 12 data sets from six studies have investigated the impact of HMB supplementation on strength index. The sample size of the included studies ranged from 113 to 433 people. Additionally, the age range of the studies was from 23 to 78 years, and the average duration of interventions was from 1 week to 1 year. Additionally, the dosage range of supplementation was from 1 to 4 g.

**TABLE 2:** Study characteristics of included studies.

First author	Year	Type of studies included	Number of studies included	Data set	Gender	Health status	Age	Dose mean (g)	Cosupplement	Duration means (week)	Outcome
Jakubowski [32]	2020	RCT	9	10	Both	Young subjects	24.21	3	Whey, glucose, Na_2_HPO_4_, K_2_PO_4_, taurine, rice flour	7.8	Body mass
											Fat mass
											Fat‐free mass
Bear (a) [33]	2019	RCT	2	2	Both	Clinical practice	78.8	3/5	NR	1	Body mass
Bear (b) [33]	2019	RCT	5	6	Both	Clinical practice	76.66	3	Vitamin D	17.80	Body mass
Bear (c) [33]	2019	RCT	3	4	Both	Clinical practice	74.52	3	Vitamin D	24	Muscle mass
Bear (d) [33]	2019	RCT	6	NR	Both	NR	56.75	3	l‐Arginine, l‐glutamine, lysine, vitamin D	22	Fat mass
Bear (e) [33]	2019	RCT	3	3	Both	Clinical practice	75.43	3	Vitamin D	14/14	Strength index
Bear (f) [33]	2019	RCT	3	3	Both	Clinical practice	70.25	3	l‐Arginine, l‐glutamine, vitamin D	13/42	Strength index
Bear (g) [33]	2019	RCT	4	4	Both	Clinical practice	60.7	3	l‐Arginine, l‐glutamine, vita mi D	14	Strength index
Sanchez‐Martinez (a) [17]	2018	RCT	6	10	Both	Trained and competitive athletes	23.3	3	l‐Carnitine, choline, boron, *Garcinia cambogia*	6/23	Body mass
Sanchez‐Martinez (b) [17]	2018	RCT	5	9	Both	Trained and competitive athletes	27.96	3	l‐Carnitine, choline, boron, *Garcinia cambogia*	7/48	Fat mass
Sanchez‐Martinez (c) [17]	2018	RCT	4	8	Both	Trained and competitive athletes	23.3	3	l‐Carnitine, choline, boron, *Garcinia cambogia*	7/75	Fat‐free mass
Sanchez‐Martinez (d) [17]	2018	RCT	4	8	Both	Trained and competitive athletes	24.8	3	NR	6/25	Strength index
Sanchez‐Martinez (e) [17]	2018	RCT	2	6	Both	Trained and competitive athletes	24.85	3	NR	5	Strength index
Holland [38]	2022	RCT	7	8	Both	Athletes	NR	3	Ca	9/25	Body mass
			5	5						10	Fat mass
			5	6						8.33	Fat‐free mass
Courel‐Ibáñez (a) [36]	2019	RCT	6	12	Both	Older adults	69.75	2.16	Vitamin D	11/13	Muscle mass
Courel‐Ibáñez (b) [36]	2019	RCT	6	8	Both	Older adults	71.01	2/5	Vitamin D, carbohydrate	11/66	Fat mass
Courel‐Ibáñez (c) [36]	2019	RCT	4	4	Both	Older adults	71	2/25	Carbohydrate, vitamin D	12/5	Strength index
Nissen [39]	2003	RCT	7	9	Both	Healthy adults	25.93	3	NR	5/2	Muscle mass
											Strength index
Wu [34]	2015	RCT	6	7	Both	Older adults	71.8	2.80	Ca, l‐arginine, l‐glutamine, lysine, resistance exercise	19.43	Muscle mass
											Fat mass
Lin (a) [37]	2021	RCT	4	4	Both	Healthy older people (exercise)	69.97	3	Carbohydrate	12	Fat mass
Lin (b) [37]	2021	RCT	4	4	Both	Healthy older people (no exercise)	73.05	2/5	l‐Arginine, l‐lysine, ascorbic acid, carbohydrate	25	
Lin (c) [37]	2021	RCT	8	8	Both	Healthy older people (total)	NR	2/75	l‐Arginine, l‐lysine, ascorbic acid, carbohydrate	18.5	
Lin (d) [37]	2021	RCT	4	4	Both	Healthy older people (exercise)	69.97	3	Carbohydrate	12	Fat‐free mass
Lin (e) [37]	2021	RCT	5	5	Both	Healthy older people (no exercise)	72.9	2/6	l‐Arginine, l‐glutamine, lysine, ascorbic acid	25/2	
Lin (f) [37]	2021	RCT	9	9	Both	Healthy older people (total)	NR	0.25	Carbohydrate, l‐arginine, l‐glutamine, lysine, ascorbic acid	18/6	
Rowlands [18]	2009	RCT	11	17	Both	Trained young	23.4	3/26	NR	5/35	Fat mass
						Untrained young					
						Trained young					Fat‐free mass
						Untrained young					
						Trained young	23				Strength index
						Untrained young					
Lin (a) [13]	2022	RCT	9	17	Both	Older adults	64.06	2/65	Vitamin D, vitamin C, l‐arginine, L‐glutamine, creatin	10/94	Strength index
Lin (b) [13]	2022	RCT	6	9	Both	Older adults	64.01	2/4	Vitamin D, vitamin C, l‐arginine, l‐glutamine, creatin	9/7	Strength index
Lin (c) [13]	2022	RCT	4	8	Both	Older adults	65.5	2/62	Vitamin D, vitamin C, l‐arginine, L‐glutamine, creatin	14/5	Strength index
Martin‐Cantero [35]	2021	RCT	3	3	Both	Older adults	73.5	2.3	NR	22/04	Muscle mass

Abbreviations: NR: not reported; RCT: randomized clinical trials.

### 3.4 The Effect of HMB on the FFM

**FIGURE 2:**
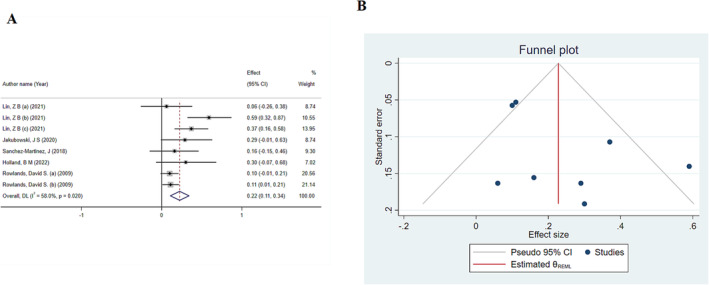
(A) The effects of HMB on fat‐free mass: Forest plot detailing effect size (ES) and 95% confidence intervals (CIs). (B) Funnel plot for the effect of HMB on fat‐free mass.

Based on the results of the analysis, HMB supplementation significantly increased FFM (ES: 0.22; 95% CI: 0.11–0.34; *p* < 0.001) (Figure 2A). Moreover, a notable level of heterogeneity between studies was observed (*I* ^2^ = 58%, *p*‐heterogeneity = 0.02). According to the results of the subgroup analysis, intervention duration was the source of heterogeneity (> 8 weeks vs. ≤ 8). Additionally, the sensitivity analysis showed that removing any of the studies did not change the final result. Moreover, the results of subgroup analyses showed that HMB in meta‐analyses in which people had an average age of ≤ 30 years resulted in a significant increase in FFM, while this effect was not significant in other age ranges. Additionally, the ES is larger in meta‐analyses that have an average intervention duration of > 8 weeks. In addition, the results of the subgroup analysis based on the average duration of interventions showed that HMB exerted a significant increase in FFM in any range of intervention durations (Table 3). The results of the publication bias analysis, which was performed based on Begg's and Egger's tests, did not show any significant results (*p* = 0.61 and 0.19). However, visual inspection of the funnel plot revealed an uneven distribution of meta‐analyses. Thus, trim‐and‐fill analysis was performed, and the results remained significant (ES: 0.22; 95% CI: 0.10–0.35) (Figure 2B).

**TABLE 3:** Subgroup analyses for the effects of HMB on body composition outcomes.

	Effect size number	ES (95% CI)^a^	*p*‐within^b^	*I* ^2^ (%)^c^	*p*‐heterogeneity^d^
Effect of HMB on strength index					
Overall	12	0.27 (0.19, 0.35)	**< 0.001**	44.4	0.04
Type of measurement					
Total	5	0.28 (0.17, 0.39)	**< 0.001**	49.6	0.09
Upper	4	0.23 (0.05, 0.42)	**0.01**	41	0.16
Lower	3	0.28 (0.04, 0.52)	**0.02**	67.8	0.04
Intervention type					
HMB	6	0.23 (0.16, 0.30)	**< 0.001**	0	0.70
HMB‐Ca	6	0.27 (0.11, 0.43)	**0.001**	64.7	0.01
Age					
≤ 30	5	0.17 (0.09, 0.25)	**< 0.001**	0	0.45
≥ 60	7	0.36 (0.29, 0.44)	**< 0.001**	0	0.56
Duration					
≤ 8	5	0.17 (0.09, 0.25)	**< 0.001**	0	0.45
> 8	7	0.36 (0.29, 0.44)	**< 0.001**	0	0.56
Quality assessment					
High quality	8	0.31 (0.20, 0.43)	**< 0.001**	45.5	0.07
Low quality	1	0.19 (−0.02, 0.40)	0.08	0	
Critically low quality	3	0.20 (0.11, 0.28)	**< 0.001**	0	0.87
Effect of HMB on fat‐free mass					
Overall	8	0.22 (0.11, 0.34)	**< 0.001**	58	0.02
Age					
≥ 70	2	0.33 (−0.19, 0.85)	0.21	83.5	0.01
NR	2	0.35 (0.17, 0.54)	**< 0.001**	0	0.75
≤ 30	4	0.12 (0.05, 0.19)	**0.001**	0	0.72
Duration					
> 8	3	0.35 (0.08, 0.62)	**0.01**	67	0.04
≤ 8	5	0.12 (0.05, 0.20)	**0.001**	0	0.70
Quality assessment					
Low quality	3	0.35 (0.08, 0.62)	**0.01**	67	0.04
High quality	2	0.22 (0.00, 0.44)	**0.04**	0	0.56
Critically low quality	3	0.11 (0.04, 0.19)	**0.001**	0	0.60
Effect of HMB on fat mass					
Overall	11	0.03 (−0.04, 0.09)	0.40	0	0.72
Age					
≥ 70	4	−0.05 (−0.21, 0.11)	0.55	0	0.77
NR	2	−0.07 (−0.28, 0.14)	0.50	0	0.39
30–50	1	0.03 (−0.28, 0.34)	0.84	0	
< 30	4	0.05 (−0.02, 0.13)	0.15	0	0.39
Duration					
> 8	7	−0.04 (−0.16, 0.07)	0.46	0	0.91
≤ 8	4	0.05 (−0.02, 0.13)	0.15	0	0.39
Quality assessment					
Low quality	4	−0.03 (−0.18, 0.12)	0.69	0	0.80
High quality	4	−0.05 (−0.19, 0.10)	0.51	0	0.55
Critically low quality	3	0.06 (−0.01, 0.14)	0.11	0	0.47
Effect of HMB on muscle mass					
Overall	5	0.21 (0.06, 0.35)	**0.004**	49.5	0.09
Age					
< 30	1	0.15 (0.06, 0.24)	**0.001**	0	
≥ 70	4	0.26 (0.02, 0.50)	**0.03**	58.7	0.06
Cosupplement					
Vitamin D	2	0.05 (−0.13, 0.24)	0.58	0	0.96
NR	2	0.30 (−0.06, 0.65)	0.10	75.9	0.04
Others	1	0.35 (0.11, 0.59)	**0.004**	0	
Duration					
>8	4	0.26 (0.02, 0.50)	**0.03**	58.7	0.06
≤8	1	0.15 (0.06, 0.24)	**0.001**	0	
Quality assessment					
Low quality	2	0.43 (0.06, 0.79)	**0.02**	12.1	0.28
High quality	2	0.19 (−0.10, 0.48)	0.20	72.9	0.5
Critically low quality	1	0.15 (0.06, 0.24)	**0.001**	0	
Effect of HMB on body mass					
Overall	5	0.09 (−0.06, 0.24)	0.22	35.1	0.18
Duration					
≤ 8	3	0.18 (−0.06, 0.42)	0.13	33.6	0.22
> 8	2	0.00 (−0.11, 0.11)	0.99	0	0.35
Quality assessment					
High quality	4	0.16 (−0.00, 0.33)	0.05	1.3	0.38
Critically low quality	1	−0.02 (−0.14, 0.10)	0.74	0	

*Note:* The bold values highlight statistically significant results (*p* < 0.05), emphasizing key findings in the subgroup analyses.

Abbreviations: CI: confidence interval; ES: effect size; NR: not reported.

^a^ Obtained from the random‐effects model.

^b^ Refers to the mean (95% CI).

^c^ Inconsistency, percentage of variation across studies due to heterogeneity.

^d^ Obtained from the *Q* test.

### 3.5 The Effect of HMB on the Strength Index

**FIGURE 3:**
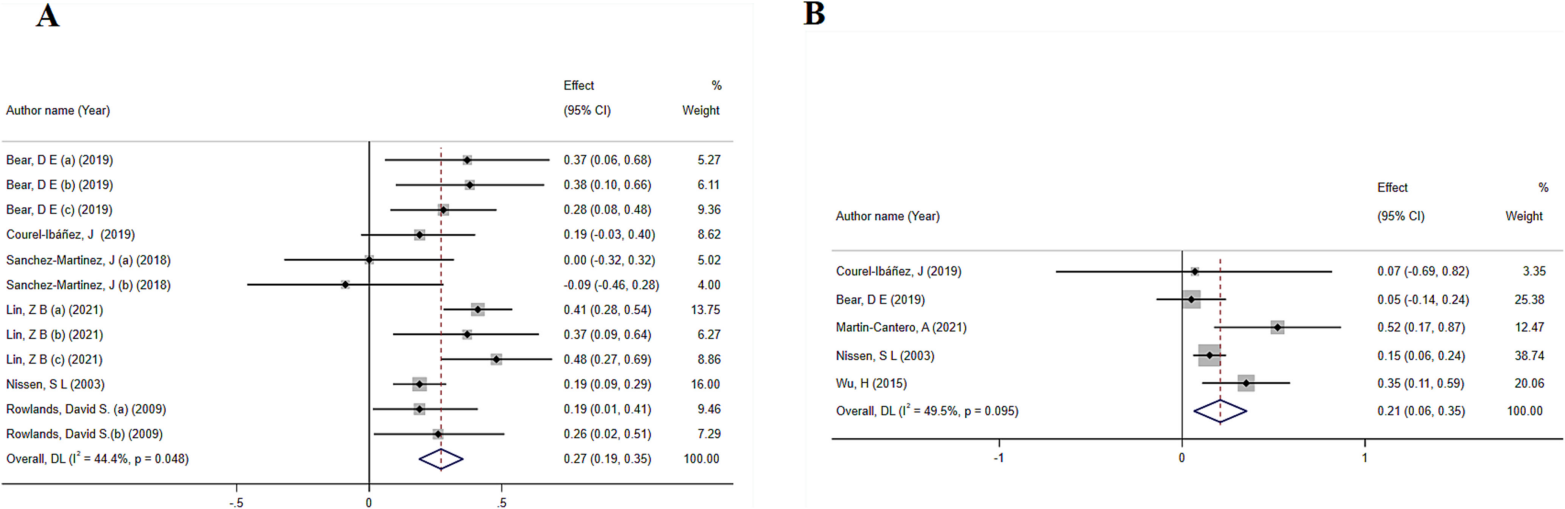
(A) The effects of HMB on strength index: Forest plot effect size (ES) and 95% confidence intervals (CIs). (B) The effects of HMB on muscle mass: Forest plot ES and 95% CIs.

The findings derived from the reanalysis of 12 data sets indicated a statistically significant increase in strength index following HMB supplementation (ES: 0.27; 95% CI: 0.19–0.35; *p* = 0.04) (Figure 3A) with a low level of heterogeneity (*I* ^2^ = 44.4%, *p*‐heterogeneity = 0.04). Additionally, the sensitivity analysis findings indicated that excluding any of the studies did not result in any changes in the overall result. The subgroup analysis revealed that HMB exhibits a more significant effect on those aged ≥ 60 years than on those ≤ 30 years of age. Additionally, research findings indicated that interventions with longer average durations (> 8 weeks) have a more beneficial effect than interventions with shorter durations (≤ 8 weeks). One of the findings from the subgroup analysis pertained to the specific type of supplement that was consumed. The findings indicated that the inclusion of calcium as a cosupplement with HMB resulted in a more pronounced impact on muscle strength compared to experiments where HMB was administered in isolation without any additional supplements. Additionally, the findings suggest that the dietary supplement exerts a more pronounced impact on the musculature of the lower body in comparison to the musculature of the upper body (Table 3). The outcomes of the analyses conducted by Begg's and Egger's tests, aimed at assessing publication bias, did not yield statistically significant results (*p* = 0.63 and 0.42).

### 3.6 The Effect of HMB on Muscle Mass

The results of the analysis of 5 included studies showed that HMB significantly increased muscle mass (ES: 0.21; 95% CI: 0.06–0.35; *p* = 0.004) (Figure 3B). The obtained heterogeneity was also within the acceptable range (*I* ^2^ = 49.5%, *p*‐heterogeneity = 0.09). Additionally, the results of the sensitivity analysis showed that removing any of the studies did not change the final result. The results of subgroup analysis regarding muscle mass showed that HMB supplementation had a larger ES in people ≥ 70 years old than in people < 30 years old. Additionally, this supplement had a greater effect in meta‐analyses with an average intervention duration of > 8 weeks compared to studies with an average intervention duration of ≤ 8 weeks.

### 3.7 The Effect of HMB on fat Mass

**FIGURE 4:**
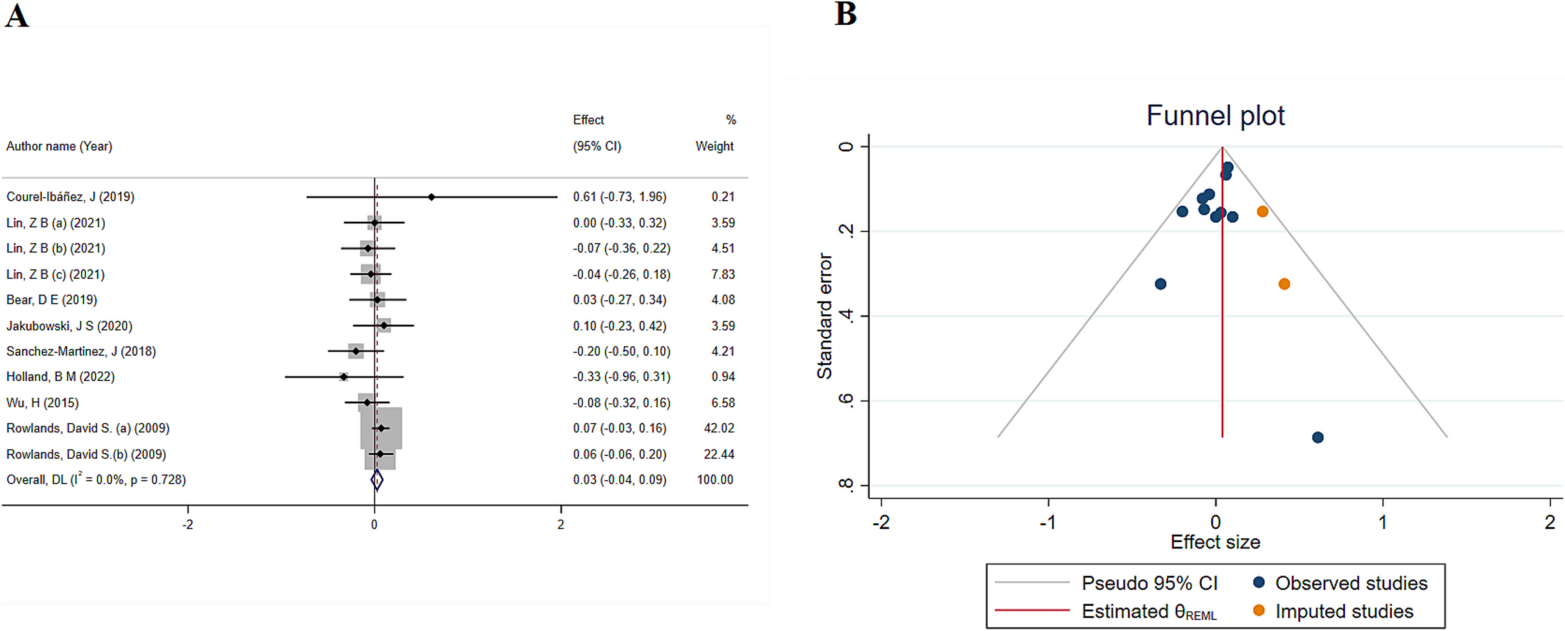
(A) The effects of HMB on fat mass: Forest plot detailing effect size (ES) and 95% confidence intervals (CIs). (B) Funnel plot for the effect of HMB on fat mass.

The findings obtained through the investigation of 11 data sets related to FM indicated that the incorporation of HMB had no statistically significant impact on this particular outcome (ES: 0.03; 95% CI: −0.04 to 0.35; *p* = 0.09) (Figure 4A). Moreover, the acquired heterogeneity remained within the permissible range (*I* ^2^ = 0.0%, *p*‐heterogeneity = 0.72). Furthermore, the findings of the sensitivity analysis indicated that the exclusion of any individual study did not result in a change in the overall result. In addition, none of the subgroup analyses based on age, duration of intervention or study quality showed statistically significant findings. Additionally, no significant small‐study effect was observed following Begg's (*p* = 0.69) and Egger's tests (*p* = 0.16). However, upon performing visual examination of the funnel plot, it was observed that the distribution of the meta‐analyses was not symmetric. Therefore, a trim‐and‐fill analysis was conducted to address this issue. However, the subsequent findings did not demonstrate any statistically significant change (ES: 0.04; 95% CI: −0.02 to 0.1, *p* > 0.05) (Figure 4B).

### 3.8 The Effect of HMB on Body Mass

**FIGURE 5:**
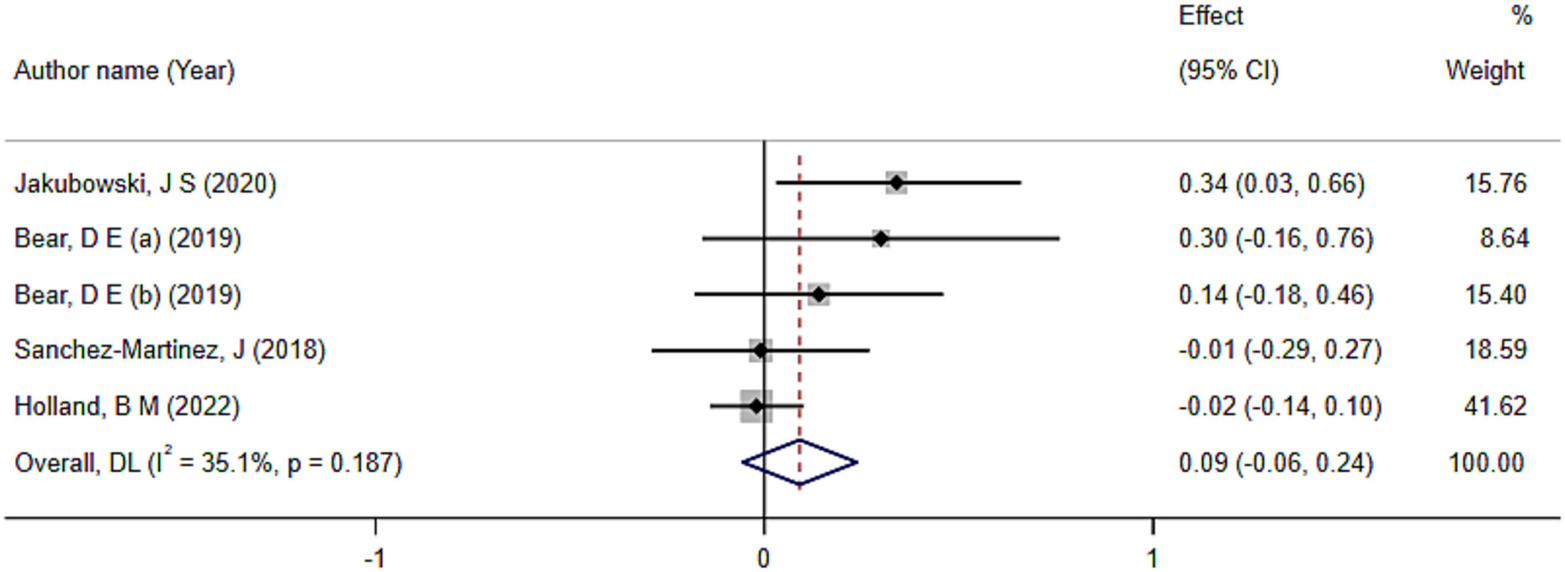
The effects of HMB on body mass: Forest plot detailing effect size (ES) and 95% confidence intervals (CIs).

Based on the results obtained from the analysis on the outcome of body mass, HMB supplementation did not have a significant effect on this outcome (ES: 0.09; 95% CI: −0.06 to 0.24; *p* = 0.22) (Figure 5). The amount of heterogeneity was low (*I* ^2^ = 35.1%, *p*‐heterogeneity = 0.18). Additionally, the results of the sensitivity analysis showed that removing any of the studies did not change the final result.

## 4 Discussion

According to the obtained results, HMB supplementation significantly increased the indices of muscle mass, muscle strength index and FFM. On the other hand, HMB consumption did not result in a significant change in FM or body mass.

HMB can improve muscle mass and the muscle strength index in a number of ways. HMB is receiving increasing attention because of its ability to stop the breakdown of proteins in skeletal muscle and to speed up protein production by turning on mechanistic target of rapamycin (mTOR) [2, 40]. Suppression of protein breakdown: HMB can also stop the breakdown of muscle proteins by blocking the ubiquitin–proteasome pathway, which is a cell process that breaks down proteins inside cells. HMB helps in the preservation of muscle protein, therefore promoting a favourable protein balance that supports the growth of muscle mass [41]. In addition, the activation and proliferation of muscle satellite cells have been observed to be stimulated by HMB. Satellite cells are a population of stem cells found within skeletal muscle tissue, fulfilling a pivotal function in the processes of muscle regeneration and growth. The promotion of satellite cell activation by HMB can increase the regeneration potential of muscle tissue [42, 43]. Also, the anti‐inflammatory actions of HMB are manifested through its ability to decrease the synthesis of proinflammatory cytokines and regulate the functioning of immune cells. The potential of HMB to reduce excessive inflammation may contribute to the facilitation of a more conducive milieu for muscle growth and recovery [44, 45]. Moreover, HMB exhibits the ability to decrease the detrimental effects of exercise‐induced muscle injury, potentially due to its antioxidative and anti‐inflammatory properties. The use of HMB in one's regimen has the potential to accelerate the recovery process and boost muscular hypertrophy by mitigating muscle injury [46, 47, 48, 49]. Furthermore, one previous study demonstrated that the administration of HMB supplements in elderly rats effectively inhibits apoptotic signalling and reduces the apoptotic index in times of muscular disuse and during subsequent reloading periods after disuse [50].

As indicated in the findings, HMB supplementation leads to a notable increase in muscle mass, FFM and muscle strength index. Additionally, it has been demonstrated that the mentioned effect becomes greater as the duration of the intervention increases. Specifically, in relation to the muscle strength index, interventions lasting more than 8 weeks on average yield an ES that is more than twice as large as interventions with an average duration of less than 8 weeks. This effect is also visible in the data derived from meta‐analytic studies. The study conducted by Bear et al. [33] and Lin et al. [13], which involved interventions lasting an average of 13 and 14 weeks, respectively, demonstrated notable positive outcomes in relation to this particular variable. In contrast, meta‐analyses with shorter average durations of intervention tend to result in ESs that are smaller or statistically insignificant. This finding is also applicable to the remaining two outcomes, indicating that studies with an intervention duration exceeding 8 weeks on average have demonstrated a threefold increase in the impact on FFM and a 1.5‐fold increase in the impact on muscle mass. Thus, it may be suggested that as the duration of the intervention increases, the impact of HMB on the three abovementioned outcomes also increases. It can be said that the long‐term use of HMB increases the FFM, reduces the breakdown of muscle protein and increases the body's recovery speed after physical activity due to the increased body's adaptation [2, 51, 52, 53]. The findings additionally indicate that the impact of HMB on muscle mass and muscle strength index is less pronounced in younger individuals compared to older individuals. However, the influence on FFM provides contrasting results. It is well acknowledged that total body mass encompasses both FM and FFM [54]. Furthermore, the proportion of FM relative to FFM tends to rise as individuals age [55]. Based on the available evidence, it can be inferred that the intake of HMB among older people leads to an increase in both muscle mass and the formation of muscle. However, it is noteworthy that the rise in FM outweighs the increase in muscle mass, therefore indicating that HMB supplementation does not provide significant effects on FFM. This may be the underlying cause of the aforementioned outcome. Furthermore, the findings from the use of HMB and calcium as a cosupplement indicate a more pronounced impact on enhancing the muscle strength index when compared to HMB supplementation alone. There are several potential factors that may contribute to this phenomenon: (1) The process of muscle contraction involves the binding of calcium ions to the protein troponin, launching a cascade of processes that facilitate the interaction between actin and myosin, the contractile proteins responsible for generating muscular force. The presence of enough calcium is essential for achieving effective muscular contraction [56]. (2) Neuromuscular function: Calcium plays a crucial role in facilitating neuromuscular transmission and mediating the intercommunication between neurons and muscles. This process enables the excretion of neurotransmitters from motor neurons, hence facilitating the stimulation and subsequent activation of muscle fibres [57]. (3) Muscle tone: Sufficient calcium levels play a crucial role in the maintenance of muscle tone, which pertains to the minimal level of tension exhibited by muscles during rest. The attainment of optimal muscle tone is associated with the enhancement of posture, stability and overall muscular strength [58]. Based on the findings obtained from the study, it can be concluded that HMB supplementation failed to have a statistically significant impact on the outcomes related to FM and body mass. One potential explanation for the lack of statistical significance in relation to the impact of body mass on the overall outcome could be attributed to the limited number of studies and data sets incorporated in the analysis, thereby resulting in a relatively smaller sample size compared to other outcomes. Among the investigations encompassed in this analysis, the findings of a study conducted by Jakubowski et al. [32], have yielded a statistically significant outcome; however, the results of the remaining studies have not demonstrated a significant effect. The potential cause of this matter is related to the type and conditions of supplementation. In conjunction with HMB, the study of Jakubowski et al. [32] incorporated whey protein, glucose, calcium and fatty acids, thereby augmenting the impact of HMB on the increase in body mass. In relation to the impact on FM, some studies included in the analysis have documented the effects of HMB supplementation on the reduction of FM, while others have observed an increase in FM. Nevertheless, it is worth noting that none of the studies used in the analysis yielded statistically significant findings. This lack of significance may have contributed to the overall conclusion also being nonsignificant. One further factor contributing to the lack of statistical significance in the overall findings is the composition of participants in the clinical trials incorporated into the meta‐analyses. It is important to note that a majority of the studies used elderly individuals. As mentioned before, there is an observed increase in the proportion of adipose tissue and FFM in older individuals. The subsequent thing to consider is that the study conducted by Rowlands and Thomson [18] showed more favourable outcomes compared to the other studies. This difference in findings may be attributed to the administration of a higher dosage of supplementary support and training during the interventions. This demonstrates that the achievement of a significant outcome can be attained by augmenting the dosage or incorporating training measures. Another important point about the muscle mass outcome is that contrary to the general result, studies by Bear et al. [33] and Courel‐Ibáñez et al. [36] have not obtained significant results, the possible reason for which could be the small number of studies entered for study Bear et al. and the low quality of study Courel‐Ibáñez et al. Additionally, regarding the FFM outcome, two studies, including Sanchez‐Martinez et al. [17] and Holland et al. [38], have not obtained significant results, contrary to the general result. The possible reason is the low number of included clinical trial studies.

## 5 Independent and Joint Effects of Exercise and HMB on Body Composition

In addition to its independent effects, HMB supplementation has been shown to work synergistically with exercise, particularly resistance training, to enhance body composition outcomes [59]. The combination of HMB and physical activity results in greater improvements in muscle strength, FFM and muscle mass compared to either intervention alone [60, 61]. HMB can enhance the anabolic response to exercise by increasing muscle protein synthesis and reducing muscle protein breakdown, making it an effective supplement for athletes and physically active individuals [62].

Studies have demonstrated that HMB, when used in conjunction with resistance training, not only improves muscle hypertrophy but also attenuates exercise‐induced muscle damage, facilitating faster recovery [46]. This dual action promoting muscle growth while reducing catabolic processes makes HMB particularly beneficial for athletes undergoing intense physical training. Additionally, the combination of HMB and exercise has been shown to have protective effects against muscle atrophy in both young and older populations, further supporting its use in improving overall body composition [36, 63].

## 6 Age‐Related Effects of HMB Supplementation on Body Composition

The effects of HMB supplementation on body composition vary significantly across different age groups, particularly between younger adults, middle‐aged adults and older adults. In younger adults (ages 18–30), the impact of HMB on muscle mass and strength appears to be less pronounced, likely due to their higher baseline levels of muscle mass and anabolic activity [64]. The minimal changes observed in FFM and strength may be attributed to the greater muscle repair and regeneration capacity typically seen in this population [65].

For middle‐aged adults (ages 30–60), HMB supplementation can have a moderate impact on muscle preservation and FFM, particularly in individuals who are physically active but not athletes [66]. As muscle protein breakdown begins to increase with age, HMB's anticatabolic effects become more relevant, though the changes are not as pronounced as in older adults [67].

In older adults (ages 60+), HMB supplementation demonstrates the most significant benefits, particularly in preventing muscle loss and increasing FFM [65]. The aging process is associated with sarcopenia and a decrease in anabolic response, making older individuals more susceptible to muscle atrophy [68, 69]. HMB's role in enhancing muscle protein synthesis and reducing muscle degradation is especially beneficial in this population, contributing to improved muscle strength and overall body composition [70]. The greater benefits observed in older adults are consistent with the findings of this review, which show that the effects of HMB on muscle mass and strength are more pronounced in this age group compared to younger individuals [71].

This distinction between age groups highlights the importance of considering age as a factor when evaluating the efficacy of HMB supplementation in different populations, particularly in the general population where fitness levels and baseline muscle mass may vary widely [41].

This umbrella review provides a novel contribution to the field by synthesizing findings from multiple meta‐analyses, rather than focusing solely on individual clinical trials or systematic reviews. While previous systematic reviews have highlighted the beneficial effects of HMB supplementation on muscle mass and strength, our review consolidates evidence across diverse study populations, intervention designs and outcome measures. This broader perspective allows us to account for variability in study designs, such as differences in participant age, physical condition and sex, and helps address inconsistencies that have been reported in previous individual studies.

Furthermore, this review highlights important gaps in the existing literature, particularly the limited number of studies for some outcomes and the heterogeneity across study populations. The findings emphasize the need for more homogenous studies and a better understanding of how different demographic groups respond to HMB supplementation. As a result, this review not only strengthens the evidence base for HMB's efficacy but also identifies areas for future research to ensure more targeted and reliable conclusions.

### 6.1 Strengths and Limitations

Based on the searches, the present study represents the first umbrella meta‐analysis investigation, focusing on the impact of HMB supplements on body composition parameters among adults. In this study, the objective was to comprehensively examine all relevant outcomes and, whenever feasible, conduct subgroup analysis. Additionally, sources of heterogeneity have been recognized, which increases the generalizability of the findings. However, there are some limitations. One of the limitations and drawbacks inherent in our investigation was the limited number of studies available for some outcomes. Consequently, this limitation may have compromised the precision of the final findings, potentially contributing to the lack of statistical significance observed in some outcomes. Another limitation of this umbrella review is the significant heterogeneity observed among the included studies, particularly in terms of the participants' age, physical condition and sex. The age range of participants spanned from 23 to 79 years, encompassing both younger adults and older individuals. Additionally, the physical condition of participants varied greatly, from untrained healthy individuals to trained athletes and clinical populations. This wide range of demographics and fitness levels complicates the generalizability of the findings. Moreover, the studies included both male and female participants, but sex was not consistently controlled for in the analyses, which may introduce bias in interpreting the results. These factors highlight the need for further research with more homogenous study populations to better understand the specific effects of HMB supplementation across different demographic groups.

## 7 Conclusion

In summary, HMB supplementation leads to a significant increase in muscle mass, FFM and muscle strength index. However, it does not have a significant impact on FM or total body mass. Therefore, HMB consumption may prove advantageous and useful for those experiencing muscular atrophy as a result of their physiological conditions. Also, the impact of HMB supplementation on muscle mass and strength appears to be more pronounced in older adults compared to younger individuals, suggesting that older populations may benefit more significantly from its ergogenic effects.

## Ethics Statement

The manuscript does not contain clinical studies or patient data.

## Conflicts of Interest

The authors declare no conflicts of interest.

## Supporting information


**Data S1** Supporting Information.

**Table S1** Results of assess the methodological quality of meta‐analysis.

## Data Availability

Data described in the manuscript will be made available upon reasonable request pending application and approval by contacting the corresponding author.
